# Modified leaky competing accumulator model of decision making with multiple alternatives: the Lie-algebraic approach

**DOI:** 10.1038/s41598-021-90356-7

**Published:** 2021-05-25

**Authors:** Chi-Fai Lo, Ho-Yan Ip

**Affiliations:** Institute of Theoretical Physics and Department of Physics, The Chinese University of Hong Kong, Shatin, N.T. Hong Kong SAR

**Keywords:** Neuroscience, Psychology, Mathematics and computing

## Abstract

In this communication, based upon the stochastic Gompertz law of population growth, we have reformulated the Leaky Competing Accumulator (LCA) model with multiple alternatives such that the positive-definiteness of evidence accumulation is automatically satisfied. By exploiting the Lie symmetry of the backward Kolmogorov equation (or Fokker–Planck equation) assoicated with the modified model and applying the Wei–Norman theorem, we have succeeded in deriving the *N*-dimensional joint probability density function (p.d.f.) and marginal p.d.f. for each alternative in closed form. With this joint p.d.f., a likelihood function can be constructed and thus model-fitting procedures become feasible. We have also demonstrated that the calibration of model parameters based upon the Monte Carlo simulated time series is indeed both efficient and accurate. Moreover, it should be noted that the proposed Lie-algebraic approach can also be applied to tackle the modified LCA model with time-varying parameters.

## Introduction

Among the current models of decision making, the *Leaky Competing Accumulator* (LCA) model^1–6^ has become fairly popular recently because it has been shown to account for a variety of behavioural datasets (mostly) related to two alternatives. In accordance with the model, evidence accumulation continues until an accumulator reaches a certain threshold level of activation, and a decision is made. Mathematically, evidence accumulation in this *N*-alternative model is described by the Ito stochastic differential equations (s.d.e.’s):

1 $$\begin{aligned} dx_{i}&= \left( I_{i}-\kappa x_{i}-\beta \sum _{j\ne i}x_{j}\right) dt+\xi dW_{i} \nonumber \\&= \left\{ I_{i}-\left( \kappa -\beta \right) x_{i}-\beta N\left( \frac{1}{N} \sum _{j=1}^{N}x_{j}\right) \right\} dt+\xi dW_{i} \end{aligned}$$

for $x_{i}\geqslant 0$ and $i=1,2,3,\ldots ,N$. Here $dx_{i}$ is the change in activation of accumulator *i*, $I_{i}$ is the input, *dt* is the time step size, $\xi$ refers to the noise and $dW_{i}$ denotes a standard Weiner process. In addition, $\kappa x_{i}$ and $\beta \sum _{j\ne i}x_{j}$ quantify the loss of activation of accumulator *i* due to leakage (sometimes called decay) and inhibition by the other accumulators, respectively. Unfortunately, the LCA model does not have a known likelihood function^7^, and the only methods available to fit the model to data are simulation-based, i.e. the LCA model has to generate simulated data for each proposed set of parameters in order to calculate any measure of fit. Hence, model-fitting procedures are extremely slow, and a thorough investigation of model-fitting procedures, recoverability and identifiability of the LCA model has not been performed for multi-alternative cases^3^. Moreover, recent research has found that the LCA model suffers from an instability problem in parameter recovery studies so that inferences made directly on the estimated parameter values are unreliable and of little meaning when applied to real data^3–6^.

Beyond question, in addition to the complicated couplings among the set of s.d.e.’s, a major hurdle in deriving a closed-form *N*-dimensional joint probability density function (p.d.f.) is the restriction that evidence accumulation is positive definite, i.e. $\left\{ x_{i}\geqslant 0\right\}$. It has been observed that under reasonable parameter ranges (i.e. when the inputs $\left\{ I_{i}\right\}$ are not too small) the effect of neglecting the restriction is insignificant^1,8–11^ , so it is justifiable to drop the constraint. The resultant model is sometimes called the Linear LCA model^12^. The special case of two alternatives can then be modelled by an Ornstein-Ulenbeck (OU) process:

2 $$\begin{aligned} dx=\left\{ \left( I_{1}-I_{2}\right) -\left( \kappa -\beta \right) x\right\} dt+\sqrt{2}\xi dW \end{aligned}$$

where $x\equiv x_{1}-x_{2}$. The OU process is a well-known process and its properties can be easily found in the literature (see, e.g.^13^). Thus, most of current research work on decision making has focused on choices between two alternatives. It is obvious that in this OU process the leakage parameter $\kappa$ and inhibition parameter $\beta$ cannot be calibrated separately for the OU process depends upon their difference only. Likewise, in order that the current models of decision making are of relevance to real life decisions, they must be applicable to understanding decisions among more than two alternatives. Accordingly, it is the aim of this communication to propose a new reformulation of the LCA model such that not only the positive-definiteness of evidence accumulation can be fulfilled automatically but also the *N*-dimensional joint p.d.f. can be derived in closed form.

First of all, by drawing a similarity between evidence accumulation and population growth, we propose to model the evidence accumulation by a generalization of the stochastic version of the Gompertz law of population growth. If $y\left( t\right)$ is the size of the cell at time *t*, the Gompertz law models the cell growth by the equation^14–17^:

3 $$\begin{aligned} \frac{dy}{y}=\left( A_{1}-A_{2}\ln y\right) dt\quad \text {for}\quad A_{2}>0 , \end{aligned}$$

where $A_{1}$, the intrinsic growth rate of the cell, is a parameter related to the initial mitosis rate and $A_{2}$, the growth deceleration factor, is related to the antiangiogenic processes. However, it should be stressed that quite often discrepancies exist between clinical data and theoretical predictions, due to more or less intense environmental fluctuations. Thus, a better model is needed to reflect the external randomness that affects the cell growth behaviour. The simplest stochastic version of the Gompertz law can be derived via assuming that the growth deceleration factor $A_{2}$ does not change while the variability of environmental conditions induces fluctuations in the intrinsic growth rate $A_{1}$^18^. By assuming that the intrinsic growth rate varies in time according to

4 $$\begin{aligned} \theta \left( t\right) =A_{1}+\sigma \varepsilon \left( t\right) , \end{aligned}$$

where $A_{1}$ is the constant mean value of $\theta \left( t\right)$, $\sigma$ is the diffusion coefficient, and $\varepsilon \left( t\right)$ is a Gaussian white noise process, the proposed stochastic version of the Gompertz law is defined by the s.d.e.:

5 $$\begin{aligned} \frac{dy}{y}=\left( A_{1}-A_{2}\ln y\right) dt+\sigma dW , \end{aligned}$$

where *dW* denotes the standard Wiener process. By Ito’s lemma, this s.d.e. implies that the exponent $x\equiv \ln y$ follows the OU process:

6 $$\begin{aligned} dx=\left\{ \left( A_{1}-\frac{1}{2}\sigma ^{2}\right) -A_{2}x\right\} dt+\sigma dW \end{aligned}$$

with the long term mean $\left\{ A_{1}-\left( 1/2\right) \sigma ^{2}\right\} /A_{2}$. It is obvious that the positive-definiteness of the size *y* of the cell is automatically fulfilled. This stochastic Gompertz model has been popularly applied to model tumour cell growth and similate the effects of a therapy recently^19–23^. In addition, this model is commonly known as the Schwartz model for modelling the mean-reverting stochastic behaviour of commodity prices in finance^24^. Beyond question, one can readily recognise that Eq. (6) is identical to the s.d.e. describing the evidence accumulation of each alternative in the absence of inhibition by the other accumulators. As a consequence, the complementary approach of letting $x_{i}$ in Eq. (1) represent the logarithm of evidence accumulation $y_{i}$ of accumulator *i* rather than the evidence accumulation presents a simple way to deal with the positive-definiteness of evidence accumulation. By Ito’s lemma, the corresponding set of s.d.e.’s which govern the evidence accumulation of each accumulator are thus given by

7 $$\begin{aligned} \frac{dy_{i}}{y_{i}}&= \left( \tilde{I}_{i}-\kappa \ln y_{i}-\beta \sum _{j\ne i}\ln y_{j}\right) dt+\xi dW_{i} \nonumber \\&= \left\{ \tilde{I}_{i}-\left( \kappa -\beta \right) \ln y_{i}-\beta N\left( \frac{1}{N}\sum _{j=1}^{N}\ln y_{j}\right) \right\} dt+\xi dW_{i} \end{aligned}$$

for $$\tilde{I}_{i}\equiv I_{i}+\left( 1/2\right) \xi ^{2}$$ and $$i=1,2,3,\ldots ,N$$.

Next, we propose a new method, namely the Lie-algebraic approach, to tackle the modified LCA model with multiple alternatives. By exploiting the Lie symmetry of the backward Kolmogorov equation (or Fokker–Planck equation) assoicated with the model and applying the Wei–Norman theorem (see Appendix A;^25^), we have succeeded in deriving the *N*-dimensional joint p.d.f. and marginal p.d.f. for each alternative in closed form. Then, a likelihood function can be constructed and model-fitting procedures become feasible. More importantly, the instability problem in parameter recovery is completely solved.

This paper is organized as follows. In second section the Lie-algebraic approach is applied to tackle the problem of the modified LCA model with *N* alternatives. Both the *N*-dimensional joint p.d.f. and marginal p.d.f. for each alternative are obtained in closed form. In “Numerical analysis” section some calibrated results based upon the Monte Carlo simulated time series are discussed and the final section presents the conclusion.

## Lie-algebraic approach

To derive the joint p.d.f. $P\left( \left\{ x_{i}\right\} ,t;\left\{ x_{i0}\right\} ,t_{0}\right)$ of the stochastic variables $\left\{ x_{1},x_{2},x_{3},\ldots ,x_{N}\right\}$ described by Eq. (1) without the restriction that $x_{i}\geqslant 0$ for $i=1,2,3,\ldots ,N$, we need to solve the associated multi-dimenstional backward Kolmogorov equation:

8 $$\begin{aligned} -\frac{\partial P\left( \left\{ x_{i}\right\} ,t;\left\{ x_{i0}\right\} ,t_{0}\right) }{\partial t_{0}} & = \sum _{i=1}^{N}\left\{ \frac{1}{2}\xi ^{2} \frac{\partial ^{2}P\left( \left\{ x_{i}\right\} ,t;\left\{ x_{i0}\right\} ,t_{0}\right) }{\partial x_{i0}^{2}}\right. \nonumber \\&\quad +\left. \left( I_{i}-\kappa x_{i0}-\beta \sum _{j\ne i}x_{j0}\right) \frac{ \partial P\left( \left\{ x_{i}\right\} ,t;\left\{ x_{i0}\right\} ,t_{0}\right) }{\partial x_{i0}}\right\} \nonumber \\ & = \left( \sum _{i=1}^{6}a_{i}\hat{L}_{i}\right) P\left( \left\{ x_{i}\right\} ,t;\left\{ x_{i0}\right\} ,t_{0}\right) \end{aligned}$$

subject to the condition $P\left( \left\{ x_{i}\right\} ,t;\left\{ x_{i0}\right\} ,t_{0}\rightarrow t\right) =\prod \nolimits _{i=1}^{N}\delta \left( x_{i}-x_{i0}\right)$, where

9 $$\begin{aligned} \hat{L}_{1} & = \sum _{i=1}^{N}\Delta I_{i}\frac{\partial }{\partial x_{i0}} ,\quad \hat{L}_{2}=\sum _{i=1}^{N}\frac{\partial }{\partial x_{i0}} ,\quad \hat{L}_{3}=\sum _{i=1}^{N}x_{i0}\frac{\partial }{\partial x_{i0} } \nonumber \\ \hat{L}_{4} & = \sum _{j=1}^{N}x_{j0}\sum _{i=1}^{N}\frac{\partial }{\partial x_{i0}},\quad \hat{L}_{5}=\sum _{i=1}^{N}\frac{\partial ^{2}}{\partial x_{i0}^{2}},\quad \hat{L}_{6}=\sum _{i=1}^{N}\sum _{j=1}^{N}\frac{ \partial ^{2}}{\partial x_{i0}\partial x_{j0}} \nonumber \\ \bar{I} & = \frac{1}{N}\sum _{i=1}^{N}I_{i},\quad \Delta I_{i}=I_{i}- \bar{I} ,\quad a_{1}=1,\quad a_{2}=\bar{I} \nonumber \\ a_{3} & = -\left( \kappa -\beta \right) ,\quad a_{4}=-\beta ,\quad a_{5}=\frac{1}{2}\xi ^{2},\quad a_{6}=0 . \end{aligned}$$

Introducing the backward time $\tau \equiv t-t_{0}$, Eq. (8) can be rewritten as

10 $$\begin{aligned} \frac{\partial P\left( \left\{ x_{i}\right\} ;\left\{ x_{i0}\right\} ,\tau \right) }{\partial \tau }=\left( \sum _{i=1}^{6}a_{i}\hat{L}_{i}\right) P\left( \left\{ x_{i}\right\} ;\left\{ x_{i0}\right\} ,\tau \right) \end{aligned}$$

with $P\left( \left\{ x_{i}\right\} ;\left\{ x_{i0}\right\} ,0\right) =\prod \nolimits _{i=1}^{N}\delta \left( x_{i}-x_{i0}\right)$. It is not difficult to show that the operators $\left\{ \hat{L}_{i}\right\}$ are the generators of a closed Lie algebra defined by the non-vanishing commutation relations:

11 $$\begin{aligned} \left[ \hat{L}_{1},\hat{L}_{3}\right]&\equiv \hat{L}_{1}\hat{L}_{3}-\hat{L} _{3}\hat{L}_{1}=\hat{L}_{1} \nonumber \\ \left[ \hat{L}_{2},\hat{L}_{3}\right] & = \frac{1}{N}\left[ \hat{L}_{2},\hat{L }_{4}\right] =\hat{L}_{2},\quad \left[ \hat{L}_{3},\hat{L}_{5}\right] =-2\hat{L}_{5} \nonumber \\ \left[ \hat{L}_{3},\hat{L}_{6}\right] & = \left[ \hat{L}_{4},\hat{L}_{5} \right] =\frac{1}{N}\left[ \hat{L}_{4},\hat{L}_{6}\right] =-2\hat{L} _{6} . \end{aligned}$$

The formal solution of the backward Kolmogorov equation is given by

12 $$\begin{aligned} P\left( \left\{ x_{i}\right\} ;\left\{ x_{i0}\right\} ,\tau \right) =\exp \left\{ \tau \left( \sum _{i=1}^{6}a_{i}\hat{L}_{i}\right) \right\} P\left( \left\{ x_{i}\right\} ;\left\{ x_{i0}\right\} ,0\right) \ . \end{aligned}$$

In accordance with the Wei–Norman theorem^25^, the exponential operator can be disentangled into the product form:

13 $$\begin{aligned} U\left( \tau \right) \equiv \exp \left\{ \tau \left( \sum _{i=1}^{6}a_{i}\hat{ L}_{i}\right) \right\} =\prod \limits _{i=1}^{6}\exp \left\{ b_{i}\left( \tau \right) \hat{L}_{i}\right\} \end{aligned}$$

where the functions $\left\{ b_{i}\left( \tau \right) \right\}$ are determined by solving a set of six coupled nonlinear ordinary differential equations with the conditions: $b_{i}\left( 0\right) =0$ for all *i* (see Appendix A). After some simple algebra we obtain

14 $$\begin{aligned} b_{1}\left( \tau \right) & = \frac{1}{\kappa -\beta }\left\{ e^{\left( \kappa -\beta \right) \tau }-1\right\} \nonumber \\ b_{2}\left( \tau \right) & = \frac{\bar{I}}{\kappa +\beta \left( N-1\right) } \left\{ e^{\left[ \kappa +\beta \left( N-1\right) \right] \tau }-1\right\} \nonumber \\ b_{3}\left( \tau \right) & = -\left( \kappa -\beta \right) \tau,\qquad b_{4}\left( t\right) =-\beta \tau \nonumber \\ b_{5}\left( \tau \right) & = \frac{\xi ^{2}}{4\left( \kappa -\beta \right) } \left\{ 1-e^{-2\left( \kappa -\beta \right) \tau }\right\} \nonumber \\ b_{6}\left( \tau \right) & = \frac{\xi ^{2}}{4N}\left\{ \frac{1-e^{-2\left[ \kappa +\beta \left( N-1\right) \right] \tau }}{\kappa +\beta \left( N-1\right) }-\frac{1-e^{-2\left( \kappa -\beta \right) \tau }}{\kappa -\beta }\right\} \ . \end{aligned}$$

The corresponding closed-form joint p.d.f. is then given by

15 $$\begin{aligned} P\left( \left\{ x_{i}\right\} ;\left\{ x_{i0}\right\} ,\tau \right) =\frac{1 }{\sqrt{\left( 4\pi \right) ^{N}\det \left( \varvec{\Omega }\right) }}\exp \left\{ -\frac{1}{4}\sum \limits _{i,j=1}^{N}\left( X_{i0}-x_{i}\right) \left( \varvec{\Omega }^{-1}\right) _{ij}\left( X_{j0}-x_{j}\right) \right\} \end{aligned}$$

where

16 $$\begin{aligned} X_{i0}=\left[ x_{i0}+b_{2}\left( \tau \right) e^{Nb_{4}\left( \tau \right) }+b_{1}\left( \tau \right) \Delta I_{i}+\frac{e^{Nb_{4}\left( \tau \right) }-1}{N}\sum \limits _{j=1}^{N}x_{j0}\right] e^{b_{3}\left( \tau \right) }\ . \end{aligned}$$

Here the $N\times N$ matrix $\varvec{\Omega }\left( \tau \right)$ is defined by its elements as follows:

17 $$\begin{aligned} \Omega _{ij}\left( \tau \right) =b_{5}\left( \tau \right) \delta _{ij}+b_{6}\left( \tau \right) , \end{aligned}$$

and $\varvec{\Omega }^{-1}\left( \tau \right)$ is its inverse.

Moreover, the marginal p.d.f. $p_{i}\left( x_{i};\left\{ x_{i0}\right\} ,\tau \right)$ for the stochastic variable $x_{i}$ can be obtained from the joint p.d.f. by integrating out the other $N-1$ variables:

18 $$\begin{aligned} p_{i}\left( x_{i};\left\{ x_{i0}\right\} ,\tau \right) =\int _{-\infty }^{\infty }dx_{1}\cdots \int _{-\infty }^{\infty }dx_{i-1}\int _{-\infty }^{\infty }dx_{i+1}\cdots \int _{-\infty }^{\infty }dx_{N}P\left( \left\{ x_{i}\right\} ;\left\{ x_{i0}\right\} ,\tau \right) \ . \end{aligned}$$

It should be noted that the marginal p.d.f. $p_{i}\left( x_{i};\left\{ x_{i0}\right\} ,\tau \right)$ satisfies the backward Kolmogorov equation subject to the condition $p_{i}\left( x_{i};\left\{ x_{i0}\right\} ,0\right) =\delta \left( x_{i}-x_{i0}\right)$. Thus, a more efficient way to derive $p_{i}\left( x_{i};\left\{ x_{i0}\right\} ,\tau \right)$ is to solve the backward Kolmogorov equation with the condition $p_{i}\left( x_{i};\left\{ x_{i0}\right\} ,0\right) =\delta \left( x_{i}-x_{i0}\right)$ directly as follows:

19 $$\begin{aligned} p_{i}\left( x_{i};\left\{ x_{i0}\right\} ,\tau \right) & = \prod \limits _{k=1}^{6}\exp \left\{ b_{k}\left( \tau \right) \hat{L} _{k}\right\} \delta \left( x_{i}-x_{i0}\right) \nonumber \\ & = \frac{1}{\sqrt{4\pi \left[ b_{5}\left( \tau \right) +b_{6}\left( \tau \right) \right] }}\exp \left\{ -\frac{\left( X_{i0}-x_{i}\right) ^{2}}{4 \left[ b_{5}\left( \tau \right) +b_{6}\left( \tau \right) \right] }\right\} \ . \end{aligned}$$

Consequently, the closed-form joint p.d.f. $P\left( \left\{ x_{i}\right\} ;\left\{ x_{i0}\right\} ,\tau \right)$ enables us to construct a likelihood function for the modified LCA model with multiple alternatives so that maximum-likelihood analyses can be performed to calibrate the parameters of the model.

As a final remark, it should be noted that the joint p.d.f. of the stochastic variables $\left\{ x_{i}\right\}$ can also be derived from solving the associated multi-dimensional Fokker–Planck equation. The details are shown in the Appendix B.

## Numerical analysis

The Monte Carlo method based upon the strong order 1.5 Taylor scheme^26^ is employed to generate the time series of the LCA model. That is, the simulation of evidence accumulation of the *i*th accumulator is performed in accordance with the following discretized version of the s.d.e. given in Eq. (1):

20 $$\begin{aligned} x_{i}^{t+\Delta t} & = x_{i}^{t}+a_{i}\Delta t+\xi \Delta W_{i}-\frac{1}{2} \left\{ \left( \kappa -\beta \right) a_{i}+\beta \sum _{j=1}^{N}a_{j}\right\} \left( \Delta t\right) ^{2} \nonumber \\&\quad-\xi \left\{ \left( \kappa -\beta \right) \Delta Z_{i}+\beta \sum _{j=1}^{N}\Delta Z_{j}\right\} , \end{aligned}$$

where

21 $$\begin{aligned} a_{i} & = \left\{ I_{i}-\left( \kappa -\beta \right) x_{i}-\beta \sum _{j=1}^{N}x_{j}\right\} \nonumber \\ \Delta W_{i} & = U_{i,1}\sqrt{\Delta t} \nonumber \\ \Delta Z_{i} & = \frac{1}{2}(\Delta t)^{3/2}\left( U_{i,1}+\frac{1}{\sqrt{3}} U_{i,2}\right) \end{aligned}$$

and $x_{i}^{t}$ denotes the logarithm of evidence accumulation of the *i*th accumulator at time *t*. Here $U_{i,1}$ and $U_{i,2}$ are uncorrelated random numbers drawn from a normal distribution with zero mean and unit variance whilst $\Delta Z_{i}$ is normally distributed with zero mean, variance $E((\Delta Z_{i})^{2})=\frac{1}{3}(\Delta t)^{3}$ and covariance $E(\Delta Z_{i}\Delta W_{i})=\frac{1}{2}(\Delta t)^{2}$. In order to simulate that the initial value of evidence accumulation is sufficiently close to zero, $x_{i}^{t=0}$ is always set equal to $-5$ in this study. For illustration, we have examined three different cases, namely the two-, three- and ten-alternative case, in each of which 128 simulated time series are generated. For each time series there are 20, 000 data points with $\Delta t=0.01$.

With the closed-form joint p.d.f., maximum likelihood analyses are then applied to calibrate the model parameters and check whether the actual values can be recovered. The global maximum of the log-likelihood function is determined by the Nelder–Mead simplex algorithm, and the implementation is performed by means of the “‘*fminsearch*” function of the MATLAB. To ensure convergence, we iterate the estimation until the discrepancy between the guess and estimated value is smaller than $10^{-6}$ in magnitude. Table 1 tabulates the average elapsed time for the maximum likelihood estimation per time series for the three illustrative cases. In fact, the calibration can be completed within a minute even for a large number of alternatives. The calibration is carried out using a 4.7 GHz Intel Core i7-10700K PC.

**Table 1:** The average elapsed time of calibration from 128 time series.

N	Data points	Average elapsed time of calibration per time series (s)
2	20,000	1.3783
3	20,000	1.7902
10	20,000	11.592

In Table 2 the input model parameters and the calibrated values (based upon 128 simulated time series) are presented for the case of two alternatives. The corresponding standard errors and z-scores of the calibrated values are tabulated, too. It is obvious that the calibrated values are in good agreement with the exact values. Tables 3 and 4 present the same set of informations for the three- and ten-alternative case respectively, and the same observation can be made. As a result, it is beyond question that the calibration of parameters is both effcient and accurate.

**Table 2:** Calibrated results for the case of two alternatives.

	$\kappa$	$\beta$	$I_1$	$I_2$	$\xi$
Exact value	4	1	0.9	1.1	0.25
Calibrated value	4.01	0.97	0.894	1.095	0.24998
Standard error	0.10	0.10	0.026	0.026	0.00089
z-score	39.7	9.7	33.9	41.4	280.1

**Table 3:** Calibrated results for the case of three alternatives.

	$\kappa$	$\beta$	$I_{1}$	$I_{2}$	$I_{3}$	$\xi$
Exact value	4	1	0.9	1.1	0.98	0.25
Calibrated value	3.963	0.998	0.894	1.094	0.978	0.24991
Standard error	0.088	0.051	0.023	0.023	0.023	0.00073
z-score	45.0	19.6	38.8	47.2	42.9	342.5

**Table 4:** Calibrated results for the case of ten alternatives.

	$\kappa$	$\theta$	$I_1$	$I_2$	$I_3$	$I_4$	$I_5$
Exact value	4	1	0.9	1.1	0.95	1.2	0.5
Calibrated value	4.08	0.990	0.898	1.103	0.954	1.199	0.484
Standard error	0.17	0.020	0.056	0.057	0.056	0.058	0.062
z-score	24.3	48.9	16.1	19.4	17.1	20.6	7.9

	$I_6$	$I_7$	$I_8$	$I_9$	$I_{10}$	$\xi$	
Exact value	0.75	0.8	1	1	1	0.25	
Calibrated value	0.741	0.796	1.006	1.008	1.005	0.25001	
Standard error	0.057	0.056	0.056	0.056	0.056	0.00040	
z-score	13.0	14.1	17.9	18.0	17.9	631.6	

## Conclusion

Based upon the stochastic Gompertz law of population growth, we have reformulated the LCA model with multiple alternatives such that the positive-definiteness of evidence accumulation is automatically satisfied. By exploiting the Lie symmetry of the backward Kolmogorov equation (or Fokker–Planck equation) assoicated with the modified model and applying the Wei–Norman theorem, we have also succeeded in deriving the *N*-dimensional joint p.d.f. and marginal p.d.f. for each alternative in closed form. With the joint p.d.f., a likelihood function can be constructed and thus model-fitting procedures become feasible and efficient. We have also demonstrated that the calibration of model parameters based upon the Monte Carlo simulated time series is indeed both efficient and accurate. Moreover, it should be noted that the proposed Lie-algebraic approach can also be applied to tackle the modified LCA model with time-varying parameters.

## Supplementary information

Supplementary Information

## References

[1] Usher M, McClelland JL (2001). The time course of perceptual choice: The leaky, competing accumulator model. Psychol. Rev..

[2] Churchland AK, Ditterich J (2012). New advances in understanding decisions among multiple alternatives. Curr. Opin. Neurobiol..

[3] Miletić S, Turner BM, Forstmann BU, Van Maanen L (2017). Parameter recovery for the leaky competing accumulator model. J. Math. Psychol..

[4] Evans NJ (2019). A method, framework and tutorial for efficiently simulating models of decision-making. Behav. Res. Methods.

[5] Evans NJ, Holmes WR, Trueblood JS (2019). Response-time data provide critical constraints on dynamic models of multi-alternative, multi-attribute choice. Psychon. Bull. Rev..

[6] Evans NJ, Dutilh G, Wagenmakers EJ, van der Maas HLJ (2020). Double responding: A new constraint for models of speeded decision making. Cogn. Psychol..

[7] Turner BM, Sederberg PB (2014). A generalized, likelihood-free method for posterior estimation. Psychol. Rev..

[8] Brown E, Holmes P (2001). Modelling a simple choice task: Stochastic dynamics of mutually inhibitory neural groups. Stoch. Dyn..

[9] McMillen T, Holmes P (2006). The dynamics of choice among multiple alternatives. J. Math. Psychol..

[10] Usher M, McClelland JL (2004). Loss aversion and inhibition in dynamical models of multialternative choice. Psychol. Rev..

[11] van Ravenzwaaij D, van der Maas HLJ, Wagenmakers EJ (2012). Optimal decision making in neural inhibition models. Psychol. Rev..

[12] Bogacz R, Usher M, Zhang J, McClelland JL (2007). Extending a biologically inspired model of choice: Multi-alternatives, nonlinearity and value-based multidimensional choice. Philos. Trans. R. Soc. B.

[13] Gardiner GW (1985). Handbook of Stochastic Methods for Physics, Chemistry and the Natural Sciences.

[14] Bass L, Green HS, Boxenbaum H (1989). Gompertzian mortality derived from competition between cell-types: Congenial, toxicologic and biometric determinants of longevity. J. Theor. Biol..

[15] Qi AS, Zheng X, Du CY, An BS (1993). A cellular automaton model of cancerous growth. J. Theor. Biol..

[16] Bassukas ID (1994). Comparative Gompertzian analysis of alterations of tumor-growth patterns. Cancer Res..

[17] Rygaard K, Spang-Thomsen M (1997). Quantitation and Gompertzian analysis of tumor growth. Breast Cancer Res. Treat..

[18] Ferrante L, Bompadre S, Possati L, Leone L (2000). Parameter estimation in a Gompertzian stochastic model for tumor growth. Biometrics.

[19] Albano G, Giorno V (2006). A stochastic model in tumor growth. J. Theor. Biol..

[20] Lo CF (2007). Stochastic Gompertz model of tumour cell growth. J. Theor. Biol..

[21] Albano G, Giorno V, Roman-Roman P, Torres-Ruiz F (2011). Inferring the effect of therapy on tumors showing stochastic Gompertzian growth. J. Theor. Biol..

[22] Moummou EK, Gutierrez R, Gutierrez-Sanchez R (2012). A stochastic Gompertz model with logarithmic therapy functions: Parameter estimation. Appl. Math. Comput..

[23] Giorno V, Spina S, Moreno-Diaz R, Pichler F, Quesada-Arencibia A (2013). A stochastic Gompertz model with jumps for an intermittent treatment in cancer growth. Computer Aided Systems Theory—EUROCAST 2013. Lecture Notes in Computer Science.

[24] Schwartz ES (1997). The stochastic behavior of commodity prices: Implications for valuation and hedging. J. Finance.

[25] Wei J, Norman E (1963). Lie algebraic solution of linear differential equations. J. Math. Phys..

[26] Kloeden RE, Platen E (1992). Numerical Solution of Stochastic Differential Equations.

