# Supplementary material for: Modified leaky competing accumulator model of decision making with multiple alternatives: the Lie-algebraic approach
Source: Sci Rep. 2021 May 25;11:10923. doi: 10.1038/s41598-021-90356-7 (PMC8149408; doi:10.1038/s41598-021-90356-7)
Supplement: Supplementary file 1 — Supplementary Information [file 41598_2021_90356_MOESM1_ESM.pdf]

Appendix A: Wei-Norman theorem

Consider the linear operator differential equation of the first order

$$\frac{d\hat{U}(t)}{dt} = \hat{H}(t)\hat{U}(t) \qquad , \qquad \hat{U}(0) = 1 \tag{A.1}$$

where $\hat{H}$ and $\hat{U}$ are both time-dependent linear operators in a Banach space or a finite-dimensional space. According to the Wei-Norman theorem [25], if the operator $\hat{H}$ can be expressed as

$$\hat{H}(t) = \sum_{n=1}^{N} a_n(t)\hat{L}_n \tag{A.2}$$

where a_n's are scalar functions of time and $\hat{L}_n$'s are the generators of an N-dimensional solvable Lie algebra or a real split 3-dimensional simple Lie algebra, then the operator $\hat{U}$ can assume the following form

$$\hat{U}(t) = \prod_{n=1}^{N} \exp\left\{b_n(t)\hat{L}_n\right\} \quad . \tag{A.3}$$

Here the b_n's are time-dependent scalar functions to be determined. To find the b_n's, we simply substitute Eq.(A.2) and Eq.(A.3) into Eq.(A.1) to obtain

$$\begin{aligned}
\sum_{n=1}^{N} a_n(t)\hat{L}_n &= \frac{d\hat{U}(t)}{dt}\hat{U}(t)^{-1} \\
&= \frac{db_1(t)}{dt}\hat{L}_1 + \frac{db_2(t)}{dt}\left\{e^{b_1(t)\hat{L}_1}\hat{L}_2 e^{-b_1(t)\hat{L}_1}\right\} + \\
&\quad \frac{db_3(t)}{dt}e^{b_1(t)\hat{L}_1}\left\{e^{b_2(t)\hat{L}_2}\hat{L}_3 e^{-b_2(t)\hat{L}_2}\right\}e^{-b_1(t)\hat{L}_1} + \\
&\quad \frac{db_4(t)}{dt}e^{b_1(t)\hat{L}_1}\left\{e^{b_2(t)\hat{L}_2}\left\{e^{b_3(t)\hat{L}_3}\hat{L}_4 e^{-b_3(t)\hat{L}_3}\right\}e^{-b_2(t)\hat{L}_2}\right\}e^{-b_1(t)\hat{L}_1} \\
&\quad + \cdots\cdots + \frac{db_N(t)}{dt}e^{b_1(t)\hat{L}_1}\left\{\cdots\left\{e^{b_{N-2}(t)\hat{L}_{N-2}}\right.\right. \\
&\quad \left.\left.\left\{e^{b_{N-1}(t)\hat{L}_{N-1}}\hat{L}_N e^{-b_{N-1}(t)\hat{L}_{N-1}}\right\}e^{-b_{N-2}(t)\hat{L}_{N-2}}\right\}\cdots\right\}e^{-b_1(t)\hat{L}_1}
\end{aligned} \tag{A.4}$$

where

$$\begin{aligned}
e^{b_j(t)\hat{L}_j}\hat{L}_k e^{-b_j(t)\hat{L}_j} &= \hat{L}_k + \frac{b_j(t)}{1!}\left[\hat{L}_j, \hat{L}_k\right] + \frac{b_j(t)^2}{2!}\left[\hat{L}_j, \left[\hat{L}_j, \hat{L}_k\right]\right] + \\
&\quad \frac{b_j(t)^3}{3!}\left[\hat{L}_j, \left[\hat{L}_j, \left[\hat{L}_j, \hat{L}_k\right]\right]\right]\cdots\cdots
\end{aligned} \tag{A.5}$$

in accordance with the Baker-Campbell-Hausdorff formula. Since the $\hat{L}_n$'s are the generators of an N-dimensional solvable Lie algebra, the right-hand side of Eq.(A.4) can be

reduced to a linear combination of the generators. Then, comparing the two sides of Eq.(A.4) term by term yields a set of coupled nonlinear differential equations as follows:

$$\frac{db_n(t)}{dt} = \sum_{m=1}^{N} \Gamma_{nm}(t)\, a_m(t) \qquad , \qquad b_n(0) = 0 \tag{A.6}$$

where Γ_{nm} are nonlinear functions of b_n's. Thus, we have transformed the linear operator differential equation in Eq.(A.1) to a set of coupled nonlinear differential equations of scalar functions in Eq.(A.6).

Given the solvable algebra defined by the non-vanishing commutation relations in Eq.(11), Eq.(A.4) can be expressed as

$$\begin{aligned}
\sum_{n=1}^{N} a_n(t)\, \hat{L}_n \;=\; & \left\{ \frac{db_1(t)}{dt} + b_1(t)\frac{db_3(t)}{dt} \right\} \hat{L}_1 + \\
& \left\{ \frac{db_2(t)}{dt} + b_2(t)\frac{db_3(t)}{dt} + N b_2(t)\frac{db_4(t)}{dt} \right\} \hat{L}_2 + \\
& \frac{db_3(t)}{dt}\hat{L}_3 + \frac{db_4(t)}{dt}\hat{L}_4 + \frac{db_5(t)}{dt} e^{-2b_3(t)}\hat{L}_5 + \\
& \left\{ e^{-2Nb_4(t)}\frac{db_6(t)}{dt} - \frac{1}{N}\left[1 - e^{-2Nb_4(t)}\right]\frac{db_5(t)}{dt} \right\} e^{-2b_3(t)}\hat{L}_6 \quad .
\end{aligned} \tag{A.7}$$

By rearranging the terms in Eq.(A.7), we obtain a set of six coupled first-order ordinary differential equations:

$$\begin{aligned}
\frac{db_1(t)}{dt} &= a_1(t) - a_3(t)\, b_1(t) \\
\frac{db_2(t)}{dt} &= a_2(t) - \{a_3(t) + N a_4(t)\}\, b_2(t) \\
\frac{db_3(t)}{dt} &= a_3(t) \\
\frac{db_4(t)}{dt} &= a_4(t) \\
\frac{db_5(t)}{dt} &= a_5(t)\, e^{2b_3(t)} \\
\frac{db_6(t)}{dt} &= \left\{ a_6(t) + \frac{a_5(t)}{N}\left[1 - e^{-2Nb_4(t)}\right] \right\} e^{2b_3(t)} e^{2Nb_4(t)} \quad .
\end{aligned} \tag{A.8}$$

For constant a_n's it is obvious that the solutions of the set of coupled ordinary differential equations can be readily derived in closed form.

Appendix B: Multi-dimensional Fokker-Planck equation

Alternatively, the joint p.d.f. $P(\{x_i\},t)$ of the stochastic variables $\{x_1, x_2, x_3, \ldots, x_N\}$ described by Eq.(1) can be derived by solving the associated multi-dimenstional Fokker-Planck equation:

$$\begin{aligned}\frac{\partial P(\{x_i\},t)}{\partial t} &= \sum_{i=1}^{N} \frac{\partial}{\partial x_i}\left\{\left[\frac{1}{2}\xi^2 \frac{\partial}{\partial x_i} - \left(I_i - \kappa x_i - \beta \sum_{j\neq i} x_j\right)\right] P(\{x_i\},t)\right\} \\ &= \left(\sum_{i=1}^{6} f_i \hat{O}_i\right) P(\{x_i\},t) \qquad \text{(B.1)}\end{aligned}$$

subject to the inital condition $P(\{x_i\},0) = \prod_{i=1}^{N} \delta(x_i - x_{i0})$, where

$$\begin{aligned}\hat{O}_1 &= \sum_{i=1}^{N} \Delta I_i \frac{\partial}{\partial x_i} \quad , \quad \hat{O}_2 = \sum_{i=1}^{N} \frac{\partial}{\partial x_i} \quad , \quad \hat{O}_3 = \sum_{i=1}^{N} \frac{\partial}{\partial x_i} x_i \\ \hat{O}_4 &= \sum_{i=1}^{N} \frac{\partial}{\partial x_i} \sum_{j=1}^{N} x_j \quad , \quad \hat{O}_5 = \sum_{i=1}^{N} \frac{\partial^2}{\partial x_i^2} \quad , \quad \hat{O}_6 = \sum_{i=1}^{N}\sum_{j=1}^{N} \frac{\partial^2}{\partial x_i \partial x_j} \\ \bar{I} &= \frac{1}{N}\sum_{i=1}^{N} I_i \quad , \quad \Delta I_i = I_i - \bar{I} \quad , \quad f_1 = -1 \quad , \quad f_2 = -\bar{I} \\ f_3 &= \kappa - \beta \quad , \quad f_4 = \beta \quad , \quad f_5 = \frac{1}{2}\xi^2 \quad , \quad f_6 = 0 \quad . \qquad \text{(B.2)}\end{aligned}$$

It is not difficult to show that the operators $\left\{\hat{O}_i\right\}$ are the generators of a closed Lie algebra defined by the non-vanishing commutation relations:

$$\begin{aligned}\left[\hat{O}_1, \hat{O}_3\right] &\equiv \hat{O}_1\hat{O}_3 - \hat{O}_3\hat{O}_1 = \hat{O}_1 \\ \left[\hat{O}_2, \hat{O}_3\right] &= \frac{1}{N}\left[\hat{O}_2, \hat{O}_4\right] = \hat{O}_2 \quad , \quad \left[\hat{O}_3, \hat{O}_5\right] = -2\hat{O}_5 \\ \left[\hat{O}_3, \hat{O}_6\right] &= \left[\hat{O}_4, \hat{O}_5\right] = \frac{1}{N}\left[\hat{O}_4, \hat{O}_6\right] = -2\hat{O}_6 \quad . \qquad \text{(B.3)}\end{aligned}$$

The formal solution of the Fokker-Planck equation is given by

$$P(\{x_i\},t) = \exp\left\{t\left(\sum_{i=1}^{6} f_i \hat{O}_i\right)\right\} P(\{x_i\},0) \quad . \qquad \text{(B.4)}$$

In accordance with the Wei-Norman theorem [25], the exponential operator can be disentangled into the product form:

$$U(t) \equiv \exp\left\{t\left(\sum_{i=1}^{6} f_i \hat{O}_i\right)\right\} = \prod_{i=1}^{6} \exp\left\{g_i(t)\,\hat{O}_i\right\} \qquad \text{(B.5)}$$

where the functions $\{g_i(t)\}$ are determined by solving a set of six coupled nonlinear ordinary differential equations (see Appendix A). After some simple algebra we obtain

$$\begin{aligned}
g_1(t) &= -\frac{1}{\kappa-\beta}\left\{1-e^{-(\kappa-\beta)t}\right\} \\
g_2(t) &= -\frac{\bar{I}}{\kappa+\beta(N-1)}\left\{1-e^{-[\kappa+\beta(N-1)]t}\right\} \\
g_3(t) &= (\kappa-\beta)t \quad , \quad g_4(t)=\beta t \\
g_5(t) &= \frac{\xi^2}{4(\kappa-\beta)}\left\{e^{2(\kappa-\beta)t}-1\right\} \\
g_6(t) &= \frac{\xi^2}{4N}\left\{\frac{e^{2[\kappa+\beta(N-1)]t}-1}{\kappa+\beta(N-1)}-\frac{e^{2(\kappa-\beta)t}-1}{\kappa-\beta}\right\} .
\end{aligned} \tag{B.6}$$

The corresponding closed-form joint p.d.f. is then given by

$$P(\{x_i\},t)=\frac{1}{\sqrt{(4\pi)^N\det(\mathbf{\Omega})}}\exp\left\{-\frac{1}{4}\sum_{i,j=1}^{N}(X_i-x_{i0})\left(\mathbf{\Omega}^{-1}\right)_{ij}(X_j-x_{j0})\right\} \tag{B.7}$$

where

$$X_i=\left[x_i+g_2(t)e^{Ng_4(t)}+g_1(t)\Delta I_i+\frac{e^{Ng_4(t)}-1}{N}\sum_{j=1}^{N}x_j\right]e^{g_3(t)} . \tag{B.8}$$

Here the $N\times N$ matrix $\mathbf{\Omega}(t)$ is defined by its elements as follows:

$$\Omega_{ij}(t)=g_5(t)\delta_{ij}+g_6(t) \ , \tag{B.9}$$

and $\mathbf{\Omega}^{-1}(t)$ is its inverse.
